# A Case-Only Genome-Wide Interaction Study of Smoking and Bladder Cancer Risk: Results from the COBLAnCE Cohort

**DOI:** 10.3390/cancers15174218

**Published:** 2023-08-23

**Authors:** Maryam Karimi, Sebastian Mendez-Pineda, Hélène Blanché, Anne Boland, Céline Besse, Jean-François Deleuze, Xiang-Yu Meng, Nanor Sirab, Karine Groussard, Thierry Lebret, Julia Bonastre, Yves Allory, François Radvanyi, Simone Benhamou, Stefan Michiels

**Affiliations:** 1 Oncostat U1018 Inserm, Équipe Labellisée Ligue Contre le Cancer, Université Paris-Saclay, 94805 Villejuif, France; 2 Bureau de Biostatistique et d’Épidémiologie, Gustave Roussy, Université Paris-Saclay, 94805 Villejuif, France; 3 CEPH-Biobank, Fondation Jean Dausset-CEPH, 75010 Paris, France; 4 Centre National de Recherche en Génomique Humaine (CNRGH), CEA, Université Paris-Saclay, 91057 Evry, France; 5 CNRS UMR144, Curie Institute, 75005 Paris, France; 6 Curie Institute, CNRS, UMR144, Molecular Oncology Team, PSL Research University, 75005 Paris, France; 7 Urology, Foch Hospital, 92150 Suresnes, France; 8 UVSQ, Curie Institute, Department of Pathology, Université Paris-Saclay, 92210 Saint-Cloud, France

**Keywords:** bladder cancer, genome-wide association study, smoking, case-only design

## Abstract

**Simple Summary:**

Bladder cancer is one of the most common cancers worldwide. The most important known cause of bladder cancer is tobacco smoking. Several genetic variations are also linked to the risk of developing bladder cancer. In this study, we focused on how smoking behavior interacts with genes to affect bladder cancer risk using the French national prospective COBLAnCE cohort (COhort to study BLAdder CancEr). The COBLAnCE cohort comprises approximately 1700 bladder cancer patients with available smoking and genetic information. We identified new chromosomal regions (specifically, genes located at 4q22.1, 12p13.1, and 16p13.3) of which variations may interact with smoking behavior in relation to bladder cancer risk. These findings need to be replicated in other studies.

**Abstract:**

Bladder cancer (BC) is the 6th most common cancer worldwide, with tobacco smoking considered as its main risk factor. Accumulating evidence has found associations between genetic variants and the risk of BC. Candidate gene-environment interaction studies have suggested interactions between cigarette smoking and *NAT2*/*GSTM1* gene variants. Our objective was to perform a genome-wide association case-only study using the French national prospective COBLAnCE cohort (COhort to study BLAdder CancEr), focusing on smoking behavior. The COBLAnCE cohort comprises 1800 BC patients enrolled between 2012 and 2018. Peripheral blood samples collected at enrolment were genotyped using the Illumina Global Screening Array with a Multi-Disease drop-in panel. Genotyping data (9,719,614 single nucleotide polymorphisms (SNP)) of 1674, 1283, and 1342 patients were analyzed for smoking status, average tobacco consumption, and age at smoking initiation, respectively. A genome-wide association study (GWAS) was conducted adjusting for gender, age, and genetic principal components. The results suggest new candidate loci (4q22.1, 12p13.1, 16p13.3) interacting with smoking behavior for the risk of BC. Our results need to be validated in other case-control or cohort studies.

## 1. Introduction

Bladder cancer (BC) ranked tenth among the most common cancer types worldwide, with an estimated total of 573,278 new bladder cancer cases and 212,536 deaths due to bladder cancer in 2020 [1]. Over three-fourths of all BC cases occur in men [1].

Bladder cancer risk factors can be divided into external exposure and genetic predisposition variants. Studies have suggested an increased risk of bladder cancer associated with occupational exposure to carcinogens [2,3,4] and water contaminants [5]. However, the most important known bladder cancer risk factor is tobacco smoking [6,7,8]. It is estimated that almost 50% of bladder cancer cases are attributable to smoking [9] and can be prevented.

Previous studies suggested a potential role of genetic predisposition in bladder cancer occurrence [10]. Recent genome-wide association studies (GWAS) have been successful in identifying variants associated with bladder cancer [11,12,13,14,15]. These studies identified susceptibility loci at 3q28 (variants with the nearest gene *TP63*) [14], 4p16.3 (variants with the nearest gene *TACC3*) [13], and 8q24.21 (variants with the nearest gene *MYC*) [14]. However, most of these variants are associated with a small bladder cancer risk. To provide more insights into the biological mechanism of bladder cancer, gene-environment (GxE) interactions have been proposed. Interactions between cigarette smoking (as the major risk of bladder cancer) and N-acetyl transferase 2 (*NAT2*) and Glutathione-s-transferaseM1 (*GSTM1*) variants, the two best-known candidates, have been consistently associated with BC development [16]. Indeed, studies have shown a higher relative risk of bladder cancer due to smoking for NAT2 slow acetylators compared to NAT2 rapid/intermediate acetylators. This was justified by the fact that *NAT2* detoxifies aromatic amines (primary bladder cancer carcinogen in tobacco) [17], and hence the effect of smoking becomes limited to smokers. The GxE interaction studies are important as they help to identify potentially new loci interacting with environmental exposure that would not be revealed by the main GWAS analysis. The results of such studies can help to set up new strategies for bladder cancer prevention and control. To date, most GxE interaction studies have focused on candidate genes, such as carcinogen detoxification genes, and much less attention has been given to ‘agnostic’, free-of-candidate, GxE interactions. Two studies went further and performed these association analyses on a wider range of single nucleotide polymorphisms (SNPs) (using more than 490,000 genotyped data [18] or imputed SNPs [19]), and found some suggestive evidence of interactions (either additive or multiplicative) between smoking and new SNPs and bladder cancer risk [18], but there is still a need for more data on the potential interaction between genetic variants and smoking habits.

However, conducting a GxE interaction study could be challenging due to the size of the study and the limited power for interaction analysis. The standard approach to test the interaction when data comes from case-control studies is to use a regression model by adding an interaction term between the variables of interest (for example, genetic variants and smoking phenotypes). Using this approach to detect an interaction effect of the same magnitude as the main effect (obtained from GWAS analysis), the sample size should be at least four times the sample used for the main effects analysis [20]. In the absence of a control group, the case-only design has been proposed as an alternative approach with more power for interaction tests [21,22].

Here, we aimed to explore the genome-wide gene-smoking interactions in bladder cancer development. While previous studies have mostly focused on smoking status and tobacco consumption [18,23,24,25], we looked at three main smoking phenotypes that characterize individuals’ smoking behavior throughout their life: age at smoking initiation, smoking status (never vs. ever smokers), and average tobacco consumption. We used the COBLAnCE cohort (COhort to study BLAdder CancEr) [26], a large prospective French-based cohort containing bladder cancer patients for whom a wide range of phenotypic and environmental measures and biological samples were prospectively collected. We conducted a genome-wide gene-smoking interaction study in bladder cancer based on ~9,700,000 imputed SNPs data from ~1700 bladder cancer patients of the European ancestry population, using a case-only design framework.

## 2. Materials and Methods

### 2.1. Study Population

COBLAnCE is a multicentre cohort of bladder cancer patients (eleven public and three private hospitals). It includes newly diagnosed patients with bladder cancer who were within 1 year of their initial diagnosis and aged 18 years or older. Recruitment was performed from December 2012 to June 2018. A total of 1800 patients were enrolled in the COBLAnCE cohort. Demographic information and detailed smoking data were collected from questionnaires completed by trained nurses who asked questions of patients at baseline.

### 2.2. Smoking Phenotypes

A complete history of tobacco smoking was ascertained separately for cigarettes, cigars, and pipes. Depending on the availability of data in the COBLAnCE cohort and based on previous genetic associations in the literature, three phenotypes were selected:

Smoking status: whether the patient reported as never smoker (less than 100 cigarettes/lifetime) or ever smoker. This phenotype was considered a binary phenotype coded by 0 (never smoker) or 1 (ever smoker).

Age at smoking initiation: the age at which the patient started smoking (cigarette/pipe or cigar). This phenotype was considered a continuous phenotype and was log-transformed since it is highly skewed.

Average tobacco consumption in grams for ever-smokers: using a complete history of tobacco smoking collected separately for cigarettes, cigars, and pipes. To standardize consumption across products, we calculated product-specific lifetime consumption (in grams of tobacco) based on the International Agency for Research on Cancer estimates of average unit weights [27,28]. The average tobacco consumption was then calculated by dividing the cumulative lifetime tobacco consumption (in grams) by the overall duration of smoking and was log-transformed since this variable is highly skewed. Given its construction, this phenotype combines both tobacco consumption and the duration of smoking across a lifetime.

The analyses of the phenotypes of age at smoking initiation and average tobacco consumption excluded never smokers.

### 2.3. Genotype Data, Quality Control, and Imputation

**Figure 1:**
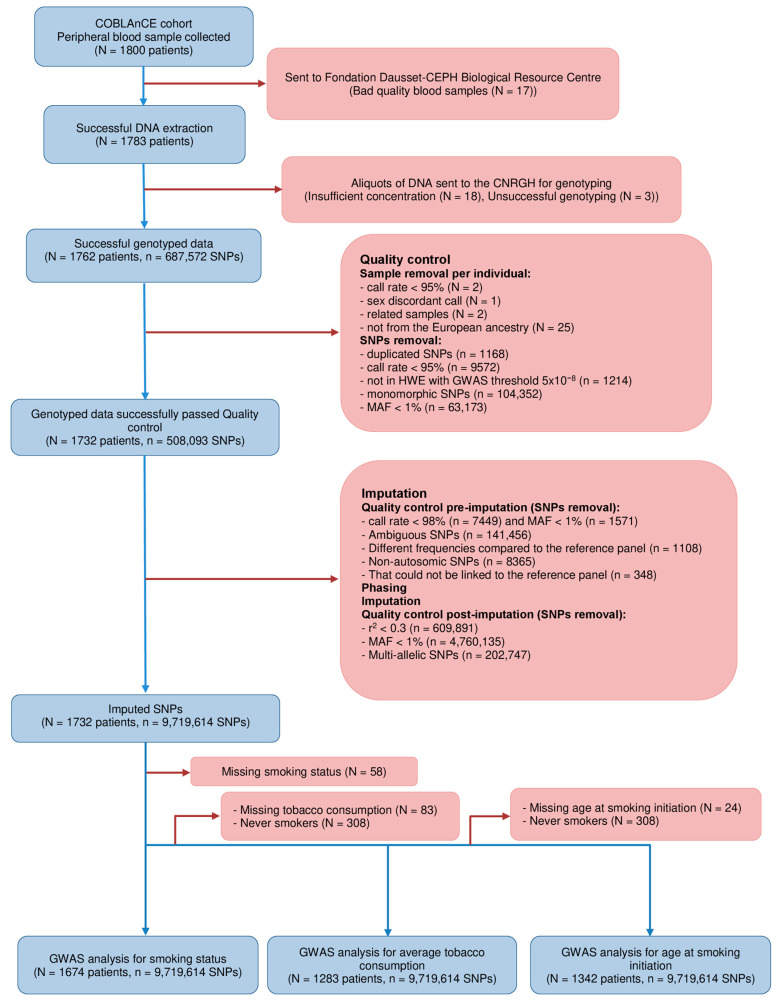
Flowchart of the study population from data collection, genotyping, and imputation to data analyses in the COBLAnCE cohort. GWAS, genome-wide association study. COBLAnCE, COhort to study BLAdder CancEr. HWE, Hardy-Weinberg Equilibrium. MAF, minor allele frequency. SNP, single nucleotide polymorphisms.

Peripheral blood samples were collected from all patients at enrolment (N = 1800). Samples were sent to the CEPH-Biobank, on average, within 48 h after collection for processing. DNA was extracted from buffy-coats using the salting-out method on the Autopure (Qiagen, Hilden, Germany) automated system and quantified using fluorimetry with the Quant-iT DNA Assay kit, Broad Range (Thermo Fisher Scientific, Waltham, MA, USA). Aliquots of DNA from 1765 patients were sent to the National Centre of Research in Human Genomics (CNRGH, CEA, Evry, France) for genotyping using the Illumina Global Screening Array with a Multi-Disease drop-in panel (GSA-MD v1.0, Illumina San Diego, CA, USA), which allows the analysis of ~700,000 polymorphisms. Genotyping was performed at the CNRGH on an Illumina automated high-throughput genotyping platform, according to the manufacturer’s instructions; 2 internal positive controls were included on each plate. All aliquoted samples were genotyped, and genotypes of 1762 patients were transferred for further analysis (failed genotyping for 21 samples, Figure 1).

Quality control (QC) on genotyped data was performed using PLINK version 1.9 [29,30]. We excluded samples with a call rate <95% (N = 2) and with a sex-discordant call (N = 1). Additionally, 2 pairs of related subjects were identified, and for each pair, the patient with the greater number of missing genotype data was excluded. Of the remaining 1757 patients, 1732 were of European ancestry. 

Of the 687,572 SNPs that were successfully obtained, we excluded duplicated SNPs (n = 1168) with a call rate <95% (n = 9572), SNPs not in Hardy-Weinberg Equilibrium (HWE) (with the GWAS threshold *p* < 5 × 10^−8^) (n = 1214), monomorphic SNPs (n = 104,352) and SNPs with a minor allele frequency (MAF) <1% (n = 63,173). The final set of SNPs retained comprised 508,093 SNPs.

Imputation of missing SNPs was performed only on the European ancestry population (N = 1732 patients and n = 508,093 SNPs), and 1000 Genome Phase 3 was used as the reference panel [31]. Additional filtering was applied to the genotyped data to increase the accuracy of the imputation. We, therefore, excluded the following SNPs from imputation: SNPs with a call rate < 98% (n = 7449) and MAF < 1% (n = 1571), ambiguous SNPs (n = 141,456), SNPs with different frequencies compared to the reference panel (n = 1108), non-autosomic SNPs (n = 8365), and SNPs that could not be linked to the reference panel (n = 348). Therefore, imputation was based on 347,796 genotyped SNPs. A two-stage approach was considered for the imputation: first, we phased GWAS samples with SHAPEIT (v2.r904) [32] using burn, prune, and main parameters (--burn 10 --prune 10 --main 50) and then genotype imputation using IMPUTE2 [33,34]. We considered non-overlapping intervals defined by the centromere locations of the human reference sequence assembly (GRCh37). For the imputation, we set the buffer region to 500 kb and the number of reference haplotypes to 800. The imputation gave us a sample of 15,292,387 SNPs. Once imputed, we excluded imputed variants with imputation info values (r^2^) < 0.3 (n = 609,891), MAF < 1% (n = 4,760,135), and multi-allelic SNPs (n = 202,747). Our final SNP population was comprised of 9,719,614 SNPs ready for use in our association analyses. 

The whole procedure (from blood collection to genotyping and imputation) is outlined in Figure 1.

### 2.4. Statistical Analysis

GWAS was performed on COBLAnCE cohort participants of European ancestry with available genotyping data after QC and imputation. We employed a case-only study design to estimate gene-environment interactions to test the association between SNPs and smoking phenotypes in bladder cancer patients. The analyses were conducted using logistic regression (for smoking status phenotype) and linear regression models (for age at smoking initiation and average tobacco consumption), with phenotype as the dependent variable against each of the imputed SNPs as the independent variable. Regressions were adjusted for gender, age, and the first ten genetic principal components to control for the study population’s latent genetic structure. The results were presented by Manhattan plots and quantile-quantile (Q-Q) plots, and the significance threshold was set at the established GWAS level (5 × 10^−8^). Functional annotation was performed for significant SNPs and the top hits using ANNOVAR [35] to identify the overlapping or closest gene to each SNP. For these SNPs, effect size estimates were presented as odds ratios (ORs), 95% confidence interval (CI) or βs, standard error (SE), and *p*-values. An OR greater than 1.0 and a β greater than 0 suggest a positive interaction for patients with reference alleles compared to those with alternative alleles. The case-only design approach relies on the independence assumption of genetic variants and environmental exposure. If the independence assumption is violated, the number of false positives will increase. A two-step approach was proposed in the literature to overcome the power insufficiency of the case-control approach [36]. In the first step, a case-only design of GxE interaction is performed, and a subset of SNPs is selected. In the second step, the selected SNPs are used in a case-control approach for the GxE interaction. This two-step strategy could help reduce false positive results. Since no control group was available in the COBLAnCE cohort, we opted for a different second step in which we considered a set of 433 SNPs from the literature. The significant association between these SNPs and different smoking phenotypes was shown within a large study of up to 1.2 million individuals [37]. We verified whether one of our top SNPs (10 SNPs with the smallest *p*-values) was part of the selected SNPs or was in high linkage disequilibrium (LD) with them

We also searched the literature for candidate SNPs that reported a smoking interaction association for bladder cancer risk (selected either from association studies with smoking phenotypes in the case-only population or from gene-smoking interaction studies in the case-control population). We narrowed our research to articles published from 2008 onwards. SNPs were filtered and retained with a reported *p* < 10^−4^ within the European population and were not associated with smoking, which led us to a total number of 37 SNPs [16,18]. We investigated the corresponding *p*-values of these SNPs in our GWAS results.

All statistical analyses were performed using PLINK v1.9 and R version 4.1.1 (https://www.r-project.org/ (accessed on 6 July 2023)).

## 3. Results

The baseline characteristics of the study population are presented in Table 1. The study population comprised 1732 patients with European ancestry (303 females and 1429 males) with a mean age of 68 years old. The difference in gender ratio was mostly attributable to the higher prevalence of smoking among males than among women in France several years ago. Accordingly, the results for smoking exposure were presented separately for males and females.

Of these 1732 patients, 308 were never smokers, and 1366 were former smokers with missing smoking status for 58 patients (3.35%). The proportion of never smokers was higher among women (37.5%) than men (13.51%).

The mean duration of tobacco consumption was almost the same for men and women (33 years vs. 34 years, respectively). However, men consumed more tobacco than women (19 g on average vs. 14 g, respectively). It seems that men started smoking earlier than women (18 years old vs. 20 years old, respectively).

**Figure 2:**
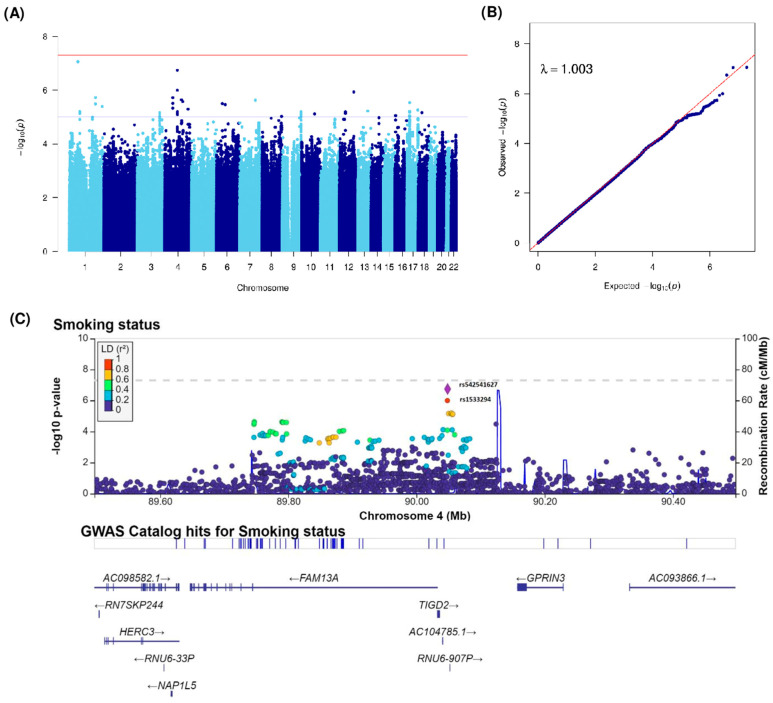
(**A**) Manhattan and (**B**) Q-Q plots of GWAS for interactions with smoking status. Blue and red horizontal lines indicate 1 × 10^−5^ and 5 × 10^−8^ (the typical GWAS threshold) thresholds, respectively. (**C**) Regional association plot for the 4q22.1 locus interacting with smoking status. The most significantly associated SNP in the region is presented in a diamond (rs542541627). Other dots correspond to other analyzed SNPs. Dots are colored by their linkage disequilibrium coefficient. GWAS, genome-wide association study. Q-Q plots, quantile–quantile plots. SNP, single nucleotide polymorphisms.

**Figure 3:**
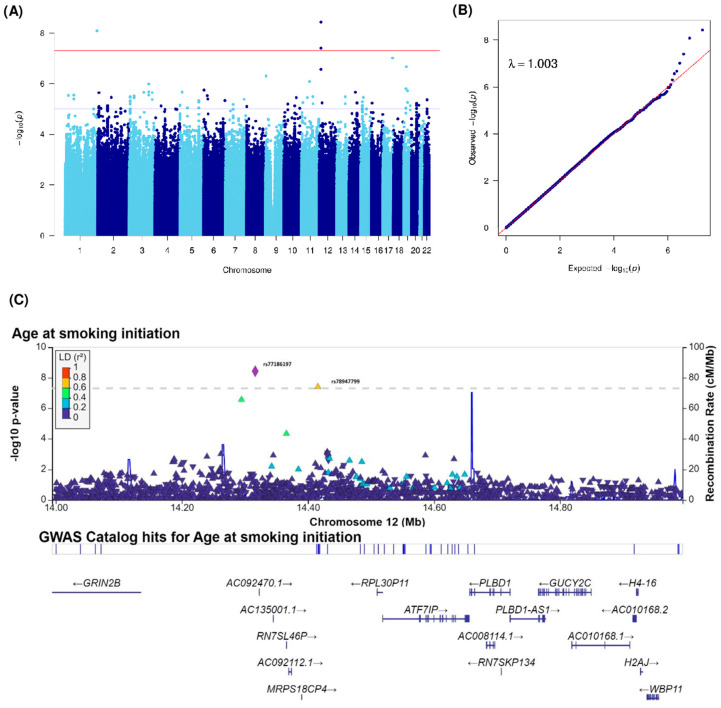
(**A**) Manhattan and (**B**) Q-Q plots of GWAS for interaction with age at smoking initiation. Blue and red horizontal lines indicate 1 × 10^−5^ and 5 × 10^−8^ (the typical GWAS threshold) thresholds, respectively. (**C**) Regional association plot for the 12p13.1 locus interacting with age at smoking initiation. The most significantly associated SNP in the region is presented in a diamond (rs77186197). Other dots correspond to other analyzed SNPs. Dots are colored by their linkage disequilibrium coefficient. GWAS, genome-wide association study. Q-Q plots, quantile–quantile plots. SNP, single nucleotide polymorphisms.

**Figure 4:**
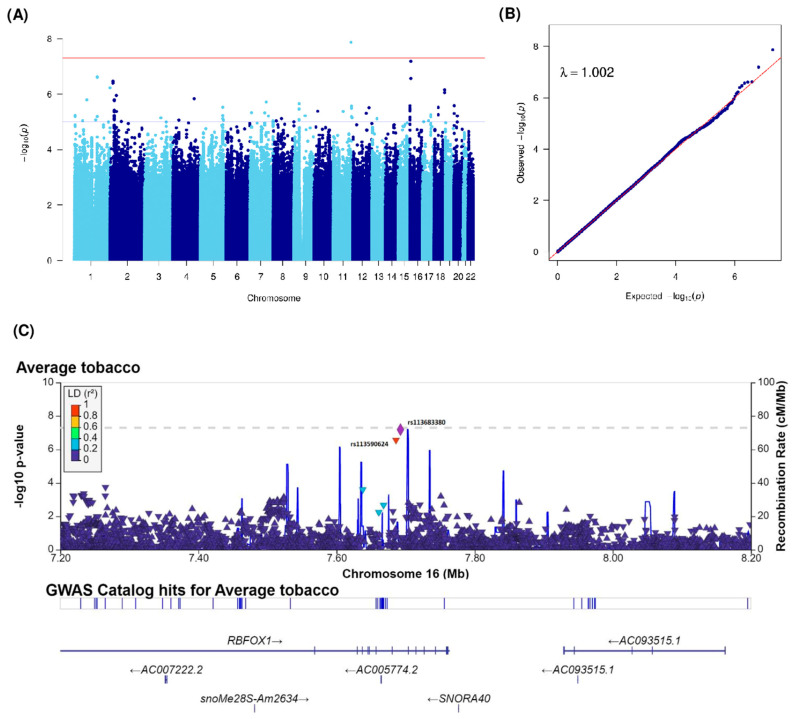
(**A**) Manhattan and (**B**) Q-Q plots of GWAS for the interaction with average tobacco consumption. Blue and red horizontal lines indicate 1 × 10^−5^ and 5 × 10^−8^ thresholds (the typical GWAS threshold), respectively; (**C**) Regional association plot for the 16p13.3 locus interacting with average tobacco consumption. The most significantly associated SNP in the region is presented in a diamond (rs113683380). Other dots correspond to other analyzed SNPs. Dots are colored by their linkage disequilibrium coefficient. GWAS, genome-wide association study. Q-Q plots, quantile-quantile plots. SNP, single nucleotide polymorphisms.

As shown in Figure 1, analyses for smoking status were carried out on 1674 patients out of 1732 patients because of the missing smoking status of 58 patients. For the age at smoking initiation and average tobacco consumption, analyses were restricted to 1342 patients and 1283 patients, respectively, because of the missing age at smoking initiation for 24 patients, having never smoker status for 308 patients, and missing tobacco consumption for 83 patients. We performed a GWAS using a case-only design. The Manhattan plot of −log10(*p*) and the Q-Q plots of the observed *p*-values versus the expected *p*-values and the genomic inflation factor (λ) of our GWAS analyses for smoking status, age at smoking initiation, and average tobacco consumption phenotypes are shown in Figure 2A,B, Figure 3A,B and Figure 4A,B. No genomic inflation was detected in our GWAS (λ = 1.003, 1.003, and 1.002 for analyses of smoking status, age at smoking initiation, and average tobacco consumption, respectively).

The top ten SNPs for each GWAS are presented in Table 2***,*** for which only four SNPs reached the genome-wide significance threshold (*p* < 5 × 10^−8^). None of these SNPs were mentioned previously in the literature in association with smoking phenotypes and were not in high LD with the 433 selected SNPs (squared correlation $r_{LD}^{2}$ between pair of SNPs ≤0.90, Supplementary Figure S1). Therefore, we presumed the independence of SNPs and smoking, which is the main hypothesis of a case-only design.

For the smoking status phenotype, none of the SNPs reached the GWAS significance threshold. However, the most significant SNP variants interacting with smoking status for BC risk were rs114073636 and rs116571608, located on chromosome 1p31.3 (OR = 0.31, 95% CI = [0.20–0.47], *p* = 8.68 × 10^−8^ and OR = 0.31, 95% CI = [0.20–0.47], *p* = 8.87 × 10^−8^, respectively for patients with reference allele compared to those with alternative allele). Both variants were intergenic. Our results also suggest a promising locus on 4q22.1 (not reaching the GWAS threshold but significant at the 10^−5^ threshold) with an intergenic SNP located between genes *TIGD2* and *GPRIN3* (Figure 2A). The regional association plot of this locus showed high LD with surrounding SNPs (Figure 2C).

Analyses yielded three SNPs significantly interacting with age at smoking initiation (Figure 3A). The reference allele at rs531756449, an intergenic variant located on chromosome 1q44, interacted positively with age at smoking initiation (β(SE) = 0.3 (0.05), *p* = 8.26 × 10^−9^). The reference allele at SNPs located on the 12p13.1 locus showed positive interaction associations with age at smoking initiation and bladder cancer risk (rs77186197 with β(SE) = 0.29 (0.05), *p* = 3.74 × 10^−9^, rs78947799 with β(SE) = 0.31 (0.06), *p* = 3.97 × 10^−8^ and rs79782126 with β(SE) = 0.18 (0.03), *p* = 2.76 × 10^−7^). The regional association plot of the 12p13.1 locus (Figure 3C) mapped between genes *GRIN2B* and *ATF7IP* shows elevated LD with two other surrounding variants (>0.4) that did not reach the significance threshold.

The reference allele of an intronic SNP, located on gene *GRAMD1B* on chromosome 11q24.1, significantly interacted positively with average tobacco consumption (rs2714069 with β(SE) = 0.63 (0.11), *p* = 1.35 × 10^−8^). However, the reference alleles of SNPs located on locus 16p13.3 seems to have protective variants for the interaction between average tobacco consumption (rs113683380 with β(SE) = −0.66 (0.12), *p* = 6.44 × 10^−8^ and rs113590624 with β(SE) = −0.64 (0.12), *p* = 2.74 × 10^−7^). These variants were intronic and located on gene *RBFOX1* (Figure 4A). The regional association plot of the 16p13.3 locus showed high LD between these two promising variants (Figure 4C).

We also investigated previously published SNPs in the literature in our GWAS results. Supplementary Table S1 shows a comparison of our results for the 37 selected SNPs. None of the SNPs were close to the GWAS threshold. However, the SNP with the smallest *p*-value was rs1495741, which showed a positive interaction with smoking initiation (OR = 1.37, 95% CI = [1.11–1.70], *p* = 0.004 for patients with reference allele compared to alternative allele), located on *NAT2*.

## 4. Discussion

The current paper focused on the interaction between SNPs and different smoking phenotypes in relation to bladder cancer risk. To our knowledge, this is the third study that focuses on free-of-candidate GxE interactions.

We performed a genome-wide interaction study of smoking phenotypes and BC risk within a case-only design framework. Our analyses were based on the COBLAnCE study, which to our knowledge, is one of the largest prospective cohorts of bladder cancer patients with numerous data and sequential biological samples collected at baseline and during the follow-up. Such prospective cohorts with large sample sizes are rare because of the major efforts required for patient recruitment and data/biomaterial collection.

Within the COBLAnCE cohort, we identified multiple loci on chromosomes 4q22.1, 12p13.1, and 16p13.3 as new candidates containing SNPs interacting with our smoking phenotypes (smoking status, age at smoking initiation and average tobacco smoking, respectively) for the risk of bladder cancer.

The reference allele at SNPs on locus 4q22.1 (rs542541627 and rs1533294) suggested a positive interaction with smoking status. These variants were intergenic and were mapped between genes *TIGD2* and *GPRIN3*. The closest gene, *TIGD2* (Tigger Transposable Element Derived 2), is a protein-coding gene. The other gene, *GPRIN3* (G Protein-Regulated Inducer of Neurite Outgrowth 3), a protein-coding gene, is predicted to be involved in neuron projection development and to be active in the plasma membrane. It has also been suggested that *GPRIN3* is associated with COPD (severe chronic obstructive pulmonary disease) and emphysema [38], and cocaine addiction [39].

The reference allele at our significant SNPs on locus 12p13.1 (rs77186197 and rs78947799) showed positive interactions with age at smoking initiation and was mapped between *GRIN2B* and *ATF7IP*. This suggests that these variants are more relevant in interaction with the early stages of smoking. The closest gene to these variants was *GRIN2B* (Glutamate Ionotropic Receptor NMDA Type Subunit 2B). A protein-coding gene that encodes a member of the N-methyl-D-aspartate (NMDA) receptor family within the ionotropic glutamate receptor superfamily. Its pathways are Transcriptional Regulation by *MECP2* (Methyl-CpG-binding protein 2) (reactome: R-HSA-8986944), which regulates neuronal receptors and channels (reactome: R-HSA-9022699), Disorders of Nervous System Development (reactome: R-HSA-9697154), and Nicotine (KEGG: mpah05033) and cocaine (KEGG: mpah05030) addiction. A new study showed that the overexpression of *MECP2* attenuates cigarette smoke extract-induced lung epithelial cell injury [40]. *GRIN2B* is also associated with nicotine dependence [41] and is one of the genes influencing smoking behaviors [42]. However, the *ATF7IP* (Activating Transcription Factor 7 Interacting Protein), a protein-coding gene, is a multifunctional nuclear protein associated with heterochromatin. *ATF7IP* is inactivated in 5% of lung adenocarcinomas among non-smokers [43].

The reference allele of two of the promising SNPs (rs113683380 and rs113590624, with a negative interaction with average tobacco consumption) located on 16p13.3 was intronic and mapped to the *RBFOX1* gene. *RBFOX1* (RNAS Binding FOX-1 Homolog 1) is a protein-coding gene that has a role in alternative splicing regulation. *RBFOX1* (also known as *A2BP1*) is highly expressed in neurons in brain regions that include the hippocampus, and there is evidence of associations between this gene, smoking cessation, and nicotine dependence [44,45]. Interestingly, one of the pathways of *RBFOX1* is transcriptional regulation by *MECP2* (reactome: R-HSA-8986944). The *RBFOX1* gene was identified as associated with substance dependence or the ability to quit smoking in 13 independent datasets [46], with smoking frequency [47]. In addition, *RBFOX1* was presented as a strong candidate for susceptibility to aggressive behavior and to several psychiatric disorders [48,49]. Our results also showed a significant interaction association with average tobacco consumption for the reference allele of an intronic SNP (rs2714069) located on the *GRAMD1B* (GRAM Domain Containing 1B) gene, a protein-coding gene. A study of breast epithelial cells showed significant upregulation in response to nicotine stress by *GRAMD1B* [50].

It is hard to compare the results of the current study with previously GxE interaction and bladder cancer risk results since most studies are based on case-control cohorts focusing only on smoking status (never vs. ever smokers). Considering a case-only design framework, our results should be interpreted as the multiplicative interaction effect of smoking, genetic variants, and the risk of bladder cancer. On the other side, most studies in the literature presented the additive interaction effect, besides two studies that also looked at the multiplicative interaction effect by performing stratified analyses [18,19]. For example, looking at the multiplicative interaction effect of *NAT2* and smoking status within a meta-analysis study [19], the same direction of the results is obtained within the COBLAnCE cohort, but the *p*-value of *NAT2* in our study did not reach the GWAS significant threshold which could be due to the small sample size compared to a meta-analysis approach.

The strength of our study comes from the COBLAnCE cohort, one of the largest prospective bladder cancer cohorts besides cohorts from Spain [51], the Netherlands [52], the UK [53], and the US [54,55]. Given the structure of the COBLAnCE cohort, it gives an accurate image of practices in bladder cancer patients in France, covering both public and private hospitals. Despite this, one of the limitations of our study is the absence of disease-free individuals. We carried out a case-only design; however, the main assumption of this approach, which is the independence between exposure and genetic variants, could not be validated in this cohort. The second limitation is the absence of replication. However, performing an exact replication study on other bladder cancer cohorts requires the availability of genotyped data and an adequate sample size, which in our case, would be smaller than the COBLAnCE cohort. Additionally, even though our cohort is one of the largest prospective bladder cancer cohorts, some SNPs had a very small MAF, and some important hits could have been lost. It is worth mentioning that we investigated the statistical interaction between genetic variants, smoking behavior, and the risk of bladder cancer. However, it does not directly imply a mechanistic interaction or causality. Our results highlight potential associations that may indicate the presence of complex biological interactions or shared pathways, but additional mechanistic studies are required to establish causality and plausible pathways.

Knowing these limitations, our results need to be investigated further in case-control or cohort studies; a GWAS meta-analysis would also be of utility to identify rare variants.

## 5. Conclusions

We found new suggestive loci on chromosomes 4q22.1, 12p13.1, and 16p13.3 interacting with different smoking phenotypes. Despite using the COBLAnCE cohort, one of the largest prospective bladder cancer cohorts, some limitations should be mentioned: use of a case-only design approach, absence of replication, and loss of information on some SNPs due to small MAF. Hence, these results need to be investigated in larger cohorts/case-control studies or within a meta-analysis framework.

## Figures and Tables

**Table 1:** Descriptive characteristics of the study population.

	Overall (N = 1732)	Women (N = 303)	Men (N = 1429)
**Age (Years)**			
Mean (SD)	68.55 (10.79)	68.05 (12.46)	68.65 (10.41)
Median (Q1, Q3)	68.70 (61.74, 76.34)	68.25 (60.27, 77.31)	68.73 (62.07, 76.10)
Min; Max	22.05; 95.31	22.2; 93.34	22.05; 95.31
Missing	1	0	1
**Smoking status**			
Never smoker	308 (17.78%)	115 (37.95%)	193 (13.51%)
Ever smoker	1366 (78.87%)	176 (58.09%)	1190 (83.28%)
Missing	58 (3.35%)	12 (3.96%)	46 (3.22%)
**Duration of smoking (Years) (N = 1366)**			
Mean (SD)	34.10 (14.54)	33.09 (14.17)	34.25 (14.59)
Median (Q1, Q3)	35.00 (24.00, 45.00)	35.00 (22.00, 44.00)	35.00 (24.00, 45.00)
Min; Max	1; 76	1; 62	1; 76
Missing	83	17	66
**Average tobacco consumption in grams (N = 1366)**			
Mean (SD)	19.82 (12.65)	16.00 (11.45)	20.36 (12.72)
Median (Q1, Q3)	18.61 (11.43, 23.51)	14.41 (9.23, 20.00)	19.11 (12.16, 24.67)
Min; Max	0.5; 115	1; 62.06	0.5; 115
Missing	83	17	66
**Age at smoking initiation (Years) (N = 1366)**			
Mean (SD)	18.40 (5.83)	20.79 (7.46)	18.06 (5.47)
Median (Q1, Q3)	18.00 (15.00, 20.00)	18 (17.00, 22.00)	17 (15.00, 20.00)
Min; Max	7; 73	10; 56	7; 73
Missing	24	5	19

SD, standard deviation; Q1, first quartile; Q3, third quartile.

**Table 2:** Top 10 SNPs of GWAS for interaction with smoking phenotypes in the COBLAnCE cohort.

Phenotype	rsID	Chr	Position	Locus	REF	ALT	MAF	OR (95% CI)	β (SE)	*p*-value	Annotation (Distance)	Type
Smoking status (Ever vs. Never)	rs114073636	1	62066340	1p31.3	G	A	0.04	0.31 (0.20–0.47)		8.68 × 10^−8^	*NFIA* (137880), *MGC34796* (53574)	intergenic
	rs116571608	1	62062694	1p31.3	G	A	0.04	0.31 (0.20–0.47)		8.87 × 10^−8^	*NFIA* (134234), *MGC34796* (57220)	intergenic
	rs2110040	1	187842047	1q31.1	G	C	0.01	0.17 (0.08–0.36)		1.88 × 10^−6^	*LINC01037* (395693)	intergenic
	rs542541627	4	90048466	4q22.1	A	AAAAACAAACAAAC	0.44	1.69 (1.39–2.06)		1.81 × 10^−7^	*TIGD2* (12414), *GPRIN3* (109068)	intergenic
	rs1533294	4	90048122	4q22.1	C	T	0.42	1.63 (1.34–1.98)		1.01 × 10^−6^	*TIGD2* (12070), *GPRIN3* (109412)	intergenic
	rs80281369	4	55856707	4q12	T	C	0.04	0.36 (0.24–0.55)		1.91 × 10^−6^	*KIT* (249826), *KDR* (87941)	intergenic
	rs11098419	4	118939953	4q26	T	G	0.30	1.62 (1.33–1.98)		2.30 × 10^−6^	*LINC02264* (148850), *NDST3* (15688)	intergenic
	rs115317515	4	129223251	4q28.2	C	T	0.02	0.29 (0.17–0.49)		2.64 × 10^−6^	*PGRMC2* (14283), *LINC02615* (125920)	intergenic
	rs76261406	7	110492301	7q31.1	T	G	0.03	0.23 (0.13–0.42)		2.39 × 10^−6^	*IMMP2L*	intronic
	rs11112182	12	105139116	12q23.3	A	G	0.19	0.57 (0.45–0.71)		1.16 × 10^−6^	*CHST11*	intronic
Age at smoking initiation (Years)	**rs531756449**	**1**	**244460577**	**1q44**	**C**	**G**	**0.02**		**0.3 (0.05)**	**8.26 × 10^−9^**	***ZBTB18* (239797), *C1orf100* (55360)**	**intergenic**
	rs140538571	3	149654460	3q25.1	C	A	0.01		−0.24 (0.05)	1.04 × 10^−6^	*RNF13*	intronic
	rs115421081	3	149600807	3q25.1	C	G	0.01		−0.24 (0.05)	1.05 × 10^−6^	*RNF13*	intronic
	rs148961658	9	4111766	9p24.2	G	C	0.01		0.26 (0.05)	5.03 × 10^−7^	*GLIS3*	intronic
	rs117818261	11	62644214	11q12.3	C	T	0.01		0.21 (0.04)	8.13 × 10^−7^	*SLC3A2*	intronic
	**rs77186197**	**12**	**14316383**	**12p13.1**	**C**	**T**	**0.01**		**0.29 (0.05)**	**3.74 × 10^−9^**	***GRIN2B* (183093), *ATF7IP*** **(202183)**	**intergenic**
	**rs78947799**	**12**	**14415811**	**12p13.1**	**A**	**C**	**0.01**		**0.31 (0.06)**	**3.97 × 10^−8^**	***GRIN2B* (282521), *ATF7IP* (102755)**	**intergenic**
	rs79782126	12	14294621	12p13.1	T	C	0.02		0.18 (0.03)	2.76 × 10^−7^	*GRIN2B* (161331), *ATF7IP* (223945)	intergenic
	rs149790626	17	72716530	17q25.1	C	T	0.01		0.35 (0.06)	9.76 × 10^−8^	*RAB37*	intronic
	rs34177209	19	18474978	19p13.11	T	A	0.26		0.06 (0.01)	1.57 × 10^−6^	*PGPEP1*	UTR3
Average tobacco consumption (grams)	rs114681930	1	160046712	1q23.2	G	T	0.01		−0.7 (0.14)	2.39 × 10^−7^	*KCNJ10* (6762), *KCNJ9* (4616)	intergenic
	rs79752468	1	160050867	1q23.2	T	A	0.01		−0.7 (0.14)	2.44 × 10^−7^	*KCNJ9* (461)	upstream
	rs142728151	1	248166173	1q44	G	A	0.02		−0.58 (0.12)	5.94 × 10^−7^	*OR2L13*	intronic
	rs114634507	2	19247230	2p24.1	G	A	0.02		−0.65 (0.13)	3.44 × 10^−7^	*LINC01376* (20504), *MIR4757* (300960)	intergenic
	rs149246142	2	19292223	2p24.1	G	A	0.02		−0.67 (0.13)	4.01 × 10^−7^	*LINC01376* (65497), *MIR4757* (255967)	intergenic
	**rs2714069**	**11**	**123389614**	**11q24.1**	**A**	**G**	**0.03**		**0.63 (0.11)**	**1.35 × 10^−8^**	***GRAMD1B***	**intronic**
	rs113683380	16	7692534	16p13.3	G	A	0.02		−0.66 (0.12)	6.44 × 10^−8^	*RBFOX1*	intronic
	rs113590624	16	7686089	16p13.3	G	C	0.02		−0.64 (0.12)	2.74 × 10^−7^	*RBFOX1*	intronic
	rs146671367	18	72146124	18q22.3	C	T	0.01		−0.92 (0.18)	6.89 × 10^−7^	*DIPK1C* (21621), *CNDP2* (17474)	intergenic
	rs151292117	18	72145473	18q22.3	G	A	0.01		−0.94 (0.19)	8.84 × 10^−7^	*DIPK1C* (20970), *CNDP2* (18125)	intergenic

ALT, alternative allele; CI, confidence interval; COBLAnCE, COhort to study BLAdder CancEr; GWAS, genome-wide association study; MAF, minor allele frequency; REF, reference allele; OR, odds ratio; rsID, SNP identification number; SNP, single nucleotide polymorphism. SNPs that are significant at the GWAS threshold are presented in bold.

## Data Availability

The GWAS summary statistics will be available in the NHGRI-EBI GWAS Catalog. Pseudoanonymised data and biological samples collected during the study can be obtained for conducting scientific projects. Requests should be made to the corresponding author and will be evaluated by the Scientific Committee of the COBLAnCE cohort. For approved projects, data transfer agreements will be required to be set up.

## Supplementary Materials

The following supporting information can be downloaded at: https://www.mdpi.com/article/10.3390/cancers15174218/s1, Figure S1: Pair-wise LD between SNPs in association with smoking phenotypes from the literature (shown in black) and the top SNPs from the COBLAnCE cohort (shown in blue). Red indicates a value of 1, and white indicates a value of 0. COBLAnCE, COhort to study BLAdder CancEr. LD, linkage disequilibrium. SNP, single nucleotide polymorphisms; Table S1: Selected SNPs from the literature and their corresponding ORs and βs in the COBLAnCE cohort.
