# Supplementary material for: A Case-Only Genome-Wide Interaction Study of Smoking and Bladder Cancer Risk: Results from the COBLAnCE Cohort
Source: Cancers (Basel). 2023 Aug 23;15(17):4218. doi: 10.3390/cancers15174218 (PMC10487226; doi:10.3390/cancers15174218)
Supplement: Supplementary file 1 [file cancers-15-04218-s001.zip › Supplementary Figure 1.pdf]

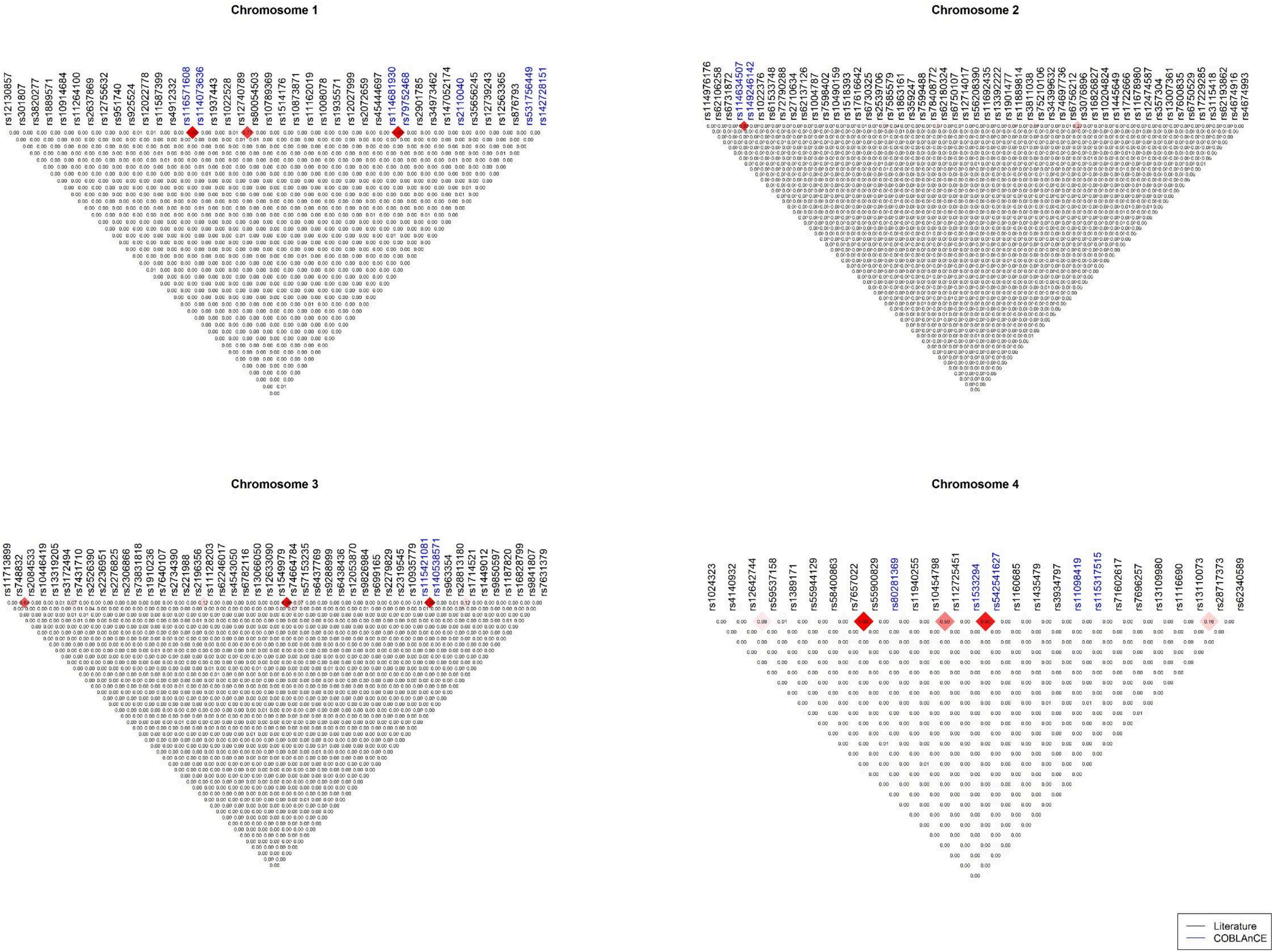
Chromosome 1
rs12130857 rs301807 rs3820277 rs1889571 rs10914684 rs11264100 rs2637869 rs12755632 rs951740 rs925524 rs12022778 rs11587399 rs4912332 rs116571608 rs114073636 rs1937443 rs1022528 rs12740789 rs80054503 rs10789369 rs1514176 rs10873871 rs11162019 rs1008078 rs1935571 rs12027999 rs2072659 rs45444697 rs114681930 rs79752468 rs2901785 rs34973462 rs147052174 rs2110040 rs35656245 rs12739243 rs12563365 rs876793 rs531756449 rs142728151
Chromosome 2
rs114976176 rs62106258 rs6731872 rs114634507 rs149246142 rs1022376 rs61533748 rs72790288 rs2710634 rs62137126 rs1004787 rs7598402 rs10490159 rs1518393 rs17616642 rs6730325 rs2539706 rs7585579 rs1863161 rs359247 rs7599488 rs78408772 rs62180324 rs6750107 rs12714017 rs56208390 rs11692435 rs13392222 rs1901477 rs11889814 rs3811038 rs75210106 rs34399632 rs74697736 rs6756212 rs3076896 rs16826827 rs10204824 rs1445649 rs1722666 rs11678980 rs12474587 rs357304 rs13007361 rs7600835 rs6750529 rs17229285 rs3115418 rs62193862 rs4674916 rs4674993
Chromosome 3
rs11713899 rs748832 rs2084533 rs10446419 rs13319205 rs3172494 rs7431710 rs2526390 rs223695 rs2276825 rs2306866 rs73831818 rs1910236 rs7640107 rs2734390 rs221988 rs2196356 rs11128203 rs62246017 rs4543050 rs6782116 rs13066050 rs12633090 rs1549979 rs74664784 rs57153235 rs6437769 rs9288999 rs6438436 rs12053870 rs9826984 rs699165 rs2279829 rs2319545 rs10935779 rs115421081 rs140538571 rs963354 rs28813180 rs1714521 rs1449012 rs9850597 rs11878820 rs16828799 rs9841807 rs7631379
Chromosome 4
rs1024323 rs4140932 rs12642744 rs59537158 rs1389171 rs55944129 rs58400863 rs7657022 rs55900829 rs80281369 rs11940255 rs10454798 rs112725451 rs1533294 rs542541627 rs1160685 rs1435479 rs3934797 rs11098419 rs115317515 rs71602617 rs7696257 rs13109980 rs1116690 rs13110073 rs28717373 rs62340589
Literature
COBLAnCE

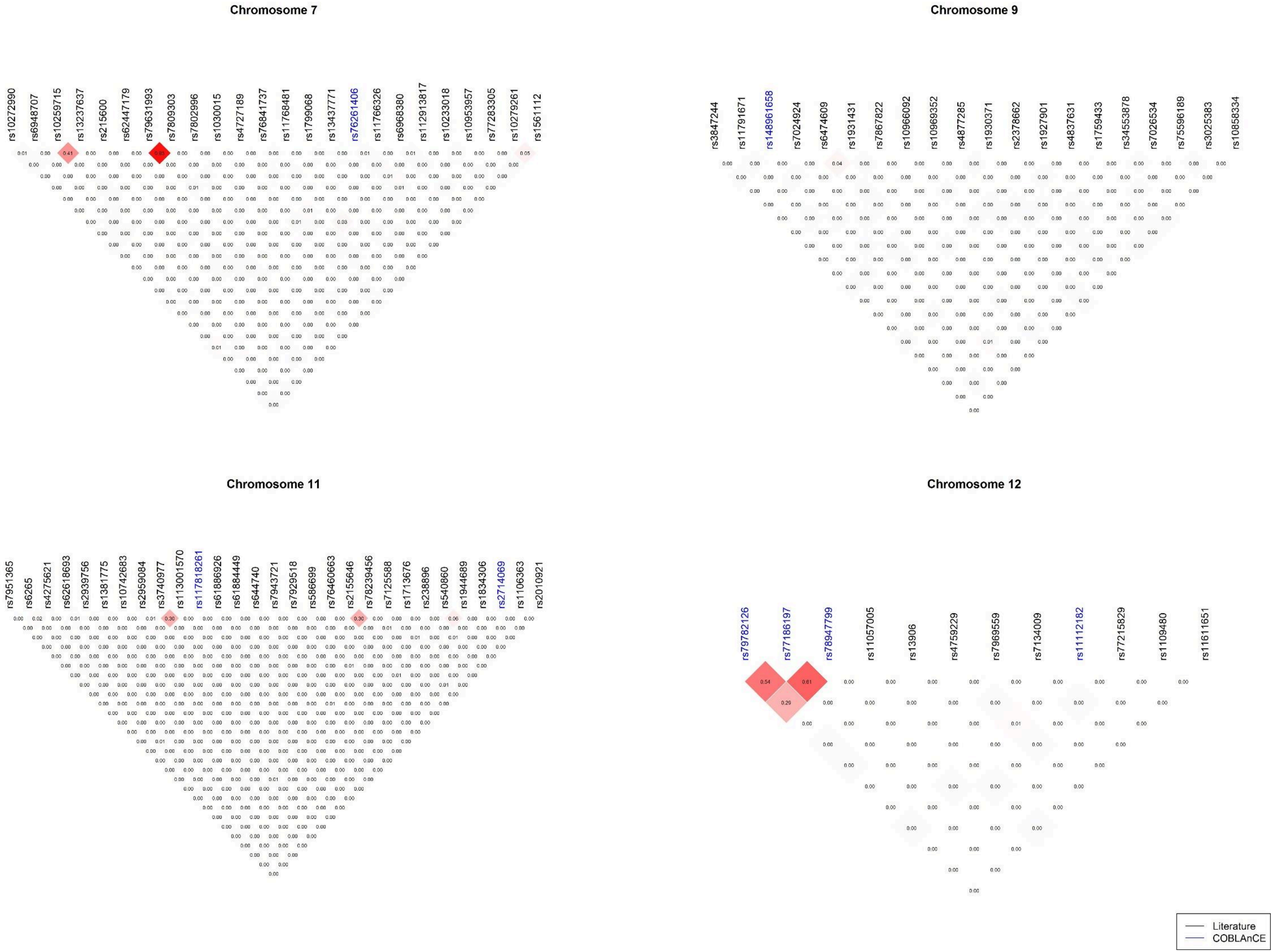
Chromosome 7
rs10272990
rs6948707
rs10259715
rs13237637
rs215600
rs62447179
rs79631993
rs7809303
rs7802996
rs1030015
rs4727189
rs76841737
rs11768481
rs1799068
rs13437771
rs76261406
rs11766326
rs6968380
rs112913817
rs10233018
rs10953957
rs77283305
rs10279261
rs1561112
Chromosome 9
rs3847244
rs11791671
rs148961658
rs7024924
rs6474609
rs1931431
rs7867822
rs10966092
rs10969352
rs4877285
rs1930371
rs2378662
rs1927901
rs4837631
rs1759433
rs34553878
rs7026534
rs75596189
rs3025383
rs10858334
Chromosome 11
rs7951365
rs6265
rs4275621
rs62618693
rs2939756
rs1381775
rs10742683
rs2959084
rs3740977
rs113001570
rs117818261
rs61886926
rs61884449
rs644740
rs7943721
rs7929518
rs586699
rs76460663
rs2155646
rs78239456
rs7125588
rs1713676
rs238896
rs540860
rs1944689
rs1834306
rs2714069
rs1106363
rs2010921
Chromosome 12
rs79782126
rs77186197
rs78947799
rs11057005
rs13906
rs4759229
rs7969559
rs7134009
rs11112182
rs77215829
rs1109480
rs11611651
Literature
COBLAnCE

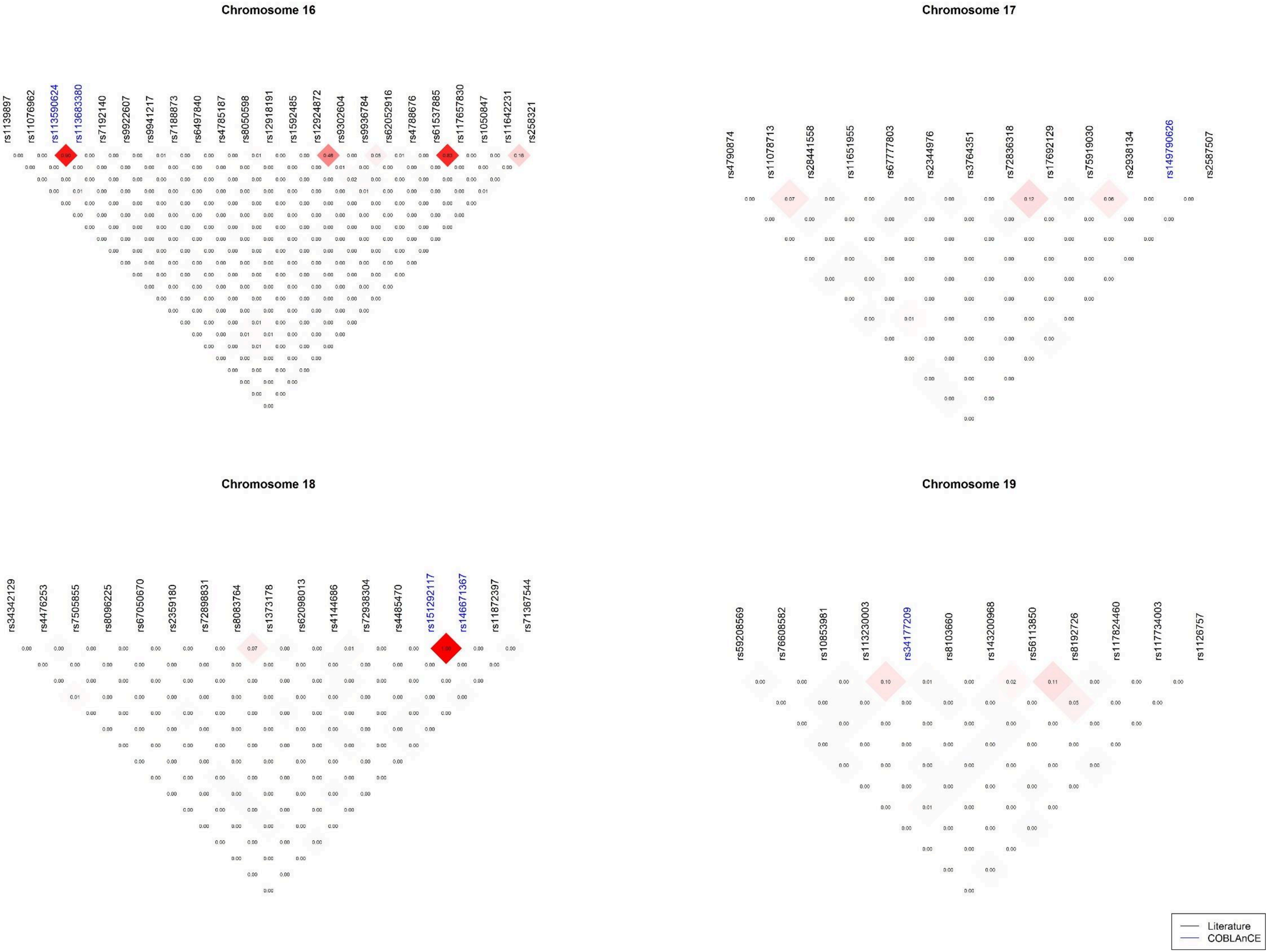
Chromosome 16
rs1139897
rs11076962
rs113590624
rs113683380
rs7192140
rs9922607
rs9941217
rs7188873
rs6497840
rs4785187
rs8050598
rs12918191
rs1592485
rs12924872
rs9302604
rs9936784
rs62052916
rs4788676
rs61537885
rs117657830
rs1050847
rs11642231
rs258321
Chromosome 17
rs4790874
rs11078713
rs28441558
rs11651955
rs67777803
rs2344976
rs3764351
rs72836318
rs17692129
rs75919030
rs2938134
rs149790626
rs2587507
Chromosome 18
rs34342129
rs4476253
rs7505855
rs8096225
rs67050670
rs2359180
rs72898831
rs8083764
rs1373178
rs62098013
rs4144686
rs72938304
rs4485470
rs151292117
rs146671367
rs11872397
rs71367544
Chromosome 19
rs59208569
rs76608582
rs10853981
rs113230003
rs34177209
rs8103660
rs143200968
rs56113850
rs8192726
rs117824460
rs117734003
rs1126757
Literature
COBLAnCE
